# Pharmacological sequestration of mitochondrial calcium uptake protects against dementia and β-amyloid neurotoxicity

**DOI:** 10.1038/s41598-022-16817-9

**Published:** 2022-07-27

**Authors:** Elena F. Shevtsova, Plamena R. Angelova, Olga A. Stelmashchuk, Noemi Esteras, Nataliia A. Vasil’eva, Andrey V. Maltsev, Pavel N. Shevtsov, Alexander V. Shaposhnikov, Vladimir P. Fisenko, Sergey O. Bachurin, Andrey Y. Abramov

**Affiliations:** 1 Institute of Physiologically Active Compounds Russian Academy of Science, Chernogolovka, Moscow Region Russia; 2 Department of Clinical and Movement Neurosciences, UCL Queen Square Institute of Neurology, London, WC1N 3BG UK; 3 Cell Physiology and Pathology Laboratory, Orel State University, Orel, Russia; 4 Department of Analysis and Forecasting of Biomedical Health Risks, Centre for Strategic Planning of FMBA of Russia, Moscow, Russia; 5 I.M. Sechenov First Moscow State Medical University, Moscow, Russia

**Keywords:** Drug screening, Cell death in the nervous system, Alzheimer's disease

## Abstract

All forms of dementia including Alzheimer’s disease are currently incurable. Mitochondrial dysfunction and calcium alterations are shown to be involved in the mechanism of neurodegeneration in Alzheimer’s disease. Previously we have described the ability of compound Tg-2112x to protect neurons via sequestration of mitochondrial calcium uptake and we suggest that it can also be protective against neurodegeneration and development of dementia. Using primary co-culture neurons and astrocytes we studied the effect of Tg-2112x and its derivative Tg-2113x on β-amyloid-induced changes in calcium signal, mitochondrial membrane potential, mitochondrial calcium, and cell death. We have found that both compounds had no effect on β-amyloid or acetylcholine-induced calcium changes in the cytosol although Tg2113x, but not Tg2112x reduced glutamate-induced calcium signal. Both compounds were able to reduce mitochondrial calcium uptake and protected cells against β-amyloid-induced mitochondrial depolarization and cell death. Behavioral effects of Tg-2113x on learning and memory in fear conditioning were also studied in 3 mouse models of neurodegeneration: aged (16-month-old) C57Bl/6j mice, scopolamine-induced amnesia (3-month-old mice), and 9-month-old 5xFAD mice. It was found that Tg-2113x prevented age-, scopolamine- and cerebral amyloidosis-induced decrease in fear conditioning. In addition, Tg-2113x restored fear extinction of aged mice. Thus, reduction of the mitochondrial calcium uptake protects neurons and astrocytes against β-amyloid-induced cell death and contributes to protection against dementia of different ethology. These compounds could be used as background for the developing of a novel generation of disease-modifying neuroprotective agents.

## Introduction

Alzheimer’s (AD) disease is the most common neurodegenerative disorder with 60–70% of all dementia cases. The major histopathological feature of AD is the deposition of extracellular senile plaques and intracellular neurofibrillary tangles containing aggregated proteins (β-amyloid, tau) and the selective loss of central cholinergic neurons^1–3^. Although molecular and cellular mechanisms of AD pathogenesis still remain unclear, the involvement of misfolded proteins, mitochondrial dysfunction and calcium deregulation in this process has been proven in various research outputs.

Several potential mechanisms of the calcium deregulation in AD have been suggested including glutamate excitotoxicity, acetylcholine receptors dysfunction and direct forming of ion channels by β-amyloid (Aβ) and tau^4–7^. Independently of the trigger of the calcium signal, elevation of Ca^2+^ in the cytosol leads to mitochondrial calcium uptake via the mitochondrial calcium uniporter (MCU)^8^. In neurons and astrocytes, mitochondrial calcium efflux is regulated by the Na^+^/Ca^2+^ exchanger NCLX. Excessive mitochondrial calcium uptake or reduced efflux might lead to mitochondrial Ca^2+^ overload, which in combination with other triggers could induce the opening of the mitochondrial permeability transition pore (mPTP), followed by cell death. Disbalance of mitochondrial Na^+^/Ca^2+^ exchange can be induced by tau as shown in cellular models^9,10^ and importantly, disfunction of mitochondrial Ca^2+^ efflux was also found in a mouse model of AD^11^. β-Amyloid is also able to induce mitochondrial calcium overload^12^ and leads to a profound mitochondrial depolarization^13^ and opening of the mPTP^14^. Importantly, cyclophilin D deficiency, simultaneously with increasing the threshold to mPTP induction, not only reduces mitochondrial and neuronal abnormality but also ameliorates learning and memory in Alzheimer's disease^15^. Additionally, increased mitochondrial calcium level was shown to be a trigger for neuronal loss in a mouse model of Alzheimer’s disease^16^.

**Figure 1:**
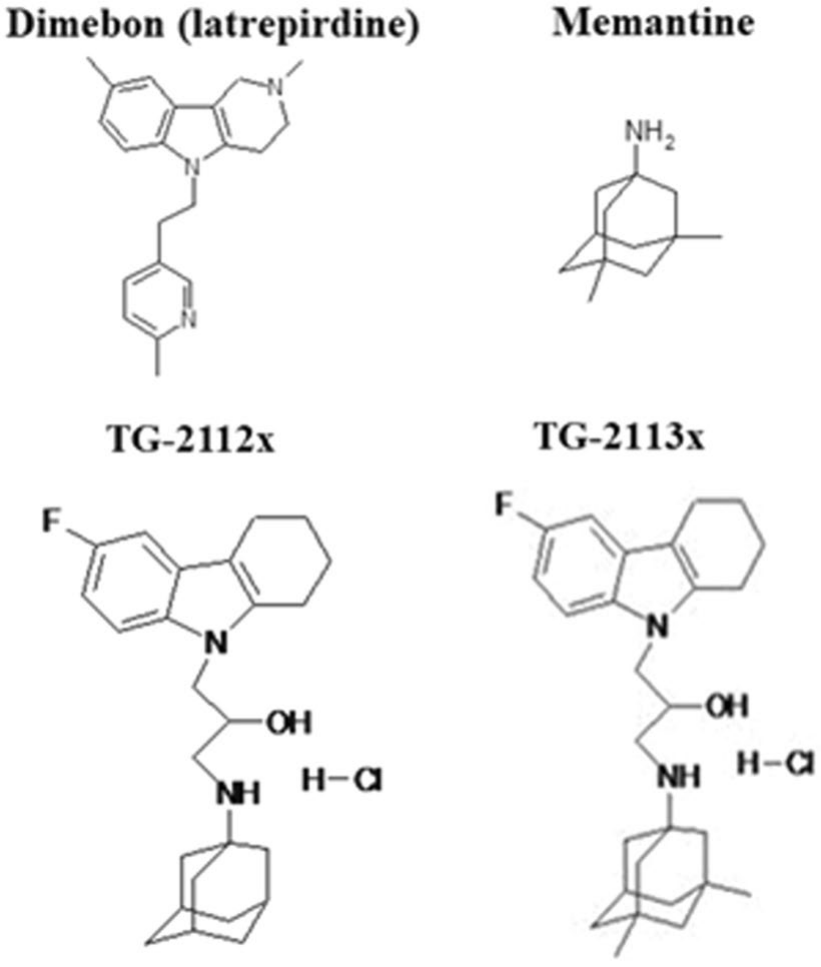
Structure of conjugates of derivatives of aminoadamantanes and ɣ-carbazoles, prototype compounds.

Recently we found that compound Tg-2112x (Fig. 1) restricted but did not completely block mitochondrial calcium uptake and protected neurons against glutamate-induced excitotoxicity^17^. Considering the importance of mitochondrial Ca^2+^ in the mechanism of neurodegeneration and dementia, in this study we used this compound and also its derivative Tg-2113x (Fig. 1)^18^, which has some advantages, i.e. affinity to glutamate receptors and microtubules stabilizing properties, to study not only how pharmacological sequestration of Ca^2+^ in mitochondria protect neurons against β-amyloid-induced cell death in primary neuronal cell cultures but also how Tg-2113x influence the memory on mouse models of dementia.

The derivatives of carbazoles, ɣ-carbolines, particularly the known neuroprotector Dimebon (Latrepirdine, Fig. 1) and derivative DF-302 have high pro-neurogenic and neuroprotective activities which are tightly connected with a mitoprotective effect^19–23^. On the other hand, Memantine (3,5-dimethyltricyclo [3.3.1.13,7] decane-1-amine, Fig. 1) is one of the approved drugs for treating dementia. Memantine also inhibit the calcium‐induced mitochondrial permeability transition and increases the calcium retention capacity of mitochondria^20,24^. In our work, Memantine, containing a free amino group, was used as a basis to design new conjugates with ɣ-carbolines and carbazoles, and among them Tg-2113x was chosen as one of the leaders according to previous in vitro studies^18^.

Following previous in vitro assays, we expected that Tg-2113x could exhibit cognition-stimulating and neuroprotective properties. Thus, Tg-2113X has been shown to increase the rate of polymerization of tubulin to form microtubules of normal structure and therefore stabilize microtubules, effectively binds to the NMDA (*N*-Methyl-d-aspartic acid) subtype of glutamate receptors, selectively inhibits butyrylcholinesterase, and increases the resistance of mitochondria to the induction of the mitochondrial permeability transition (MPT)^18^.

In the present work, we have explored the potential neuroprotective effect of Tg-2113x on cellular models of neurodegeneration with calcium overload and β-amyloid toxicity, and in in vivo models of cognitive dysfunction. For the latter, we have used three different mouse models—(1) age-related decline in cognitive function in 16-months-old C57Bl/6j mice; (2) scopolamine-induced amnesia in 3-months-old C57Bl/6j mice; and (3) transgenic cerebral amyloidosis and Alzheimer's disease model, 5xFAD mice.

We evaluated the effectiveness of Tg-2113x in aged mice, since age is considered one of the main etiological factors in the development of dementia. An important advantage of the model is the natural development of complex molecular abnormalities, which are not fully understood yet, and that lead to behavioral changes similar to the clinical signs of dementia^25^. Senile dementia is largely associated with an impairment of mitochondrial functions, in particular with a reduced threshold for induction of the mPTP, and with the disruption of cholinergic transmission^26–29^, that according to our in vitro study can be eliminated by Tg-2113x.

We chose scopolamine-induced amnesia as a model of the cholinergic impairment that often accompanies normal and pathological aging, and dementia^30^. Scopolamine is a non-selective, competitive inhibitor of muscarinic receptors and is widely used in preclinical studies for a "cholinergic" model of memory impairment^31–35^. It is believed that the amnestic effect of scopolamine can also be explained by the decreased activity of NMDA receptors^36^. The activity of glutamate receptors is important for the development of long-term potentiation (LTP), the memory formation mechanism^37^. Glutamate increases the potential to a certain level, leading to the removal of the magnesium block from the channel, are required to activate NMDA receptors. The process of increasing the potential regulates low-conductance calcium-activated potassium channels, through which the potassium ions leave the cell. Activation of M1 muscarinic receptors leads to the loss of sensitivity to calcium ions, and calcium-activated potassium channels cease to work. Scopolamine blocks the M1 receptors, and leaves the channels open, which makes it difficult to maintain the LTP, thereby causing amnesia^38^.

5xFAD is considered to be one of the most aggressive models of the hereditary form of AD or cerebral amyloidogenesis as one of the possible triggers of the senile form of AD. In these mice, the biochemical markers of dementia, such as, for example, PDAPP, Tg2576, TgAPP/Ld/2, appear 10–12 months earlier than other transgenic lines^39^. They express five mutations in human beta-amyloid precursor protein (AβPP) and presenilin (PS1, one of the four core subunits of γ-secretase) that promote the amplified production of pathological forms of β-amyloid: 3 mutations in the human APP (Swedish mutation K670N / M671L; Florida mutation I716V; and London—V717I, named for the country where the it was found) and 2 mutations in PS1 (M146L and L286V)^40^. In this model, the Swedish mutation increases the production of all Aβ, while the other four mutations increase the production of especially neurotoxic Aβ42. Thus, the simultaneous combination of many mutations leads to the formation of amyloid plaques in 1.5–2-month-old mice, and around the age of 6-months Aβ fills most of the hippocampus^41–43^.

It is known that not only associative learning, but also the extinction of the memory, i.e., suppression of irrelevant information, is important for normal cognitive functions, and this process is impaired in elderly people and patients with dementia. But while the processes of memory consolidation are widely studied and prospective therapeutic drugs are offered, the pathology in extinction processes has been much less explored, both from researchers and pharmaceutical companies. Therefore, in this paper, we used the protocol for fear conditioning, which includes, in addition to the conditioning session, the extinction session to understand the protective role of Tg-2113x (Fig. 7a).

## Methods

### Mitochondrial isolation

Rat brain non-synaptosomal mitochondria were isolated by centrifugation in Percoll gradient^21,44^. In brief: rat was euthanized by Carbon Dioxide inhalation and the brain was quickly removed, homogenized in an ice-cold isolation buffer (IB), pH 7.4: 75 mM sucrose, 225 mM mannitol, 10 mM K-HEPES with addition of 0.5 mM EGTA, 0.5 mM EDTA and 1 mg/ml BSA, and the homogenate was centrifuged for 11 min at 1500*g*. The pellet was homogenized in half of the volume of the same buffer and centrifugation was repeated. The combined supernatants were centrifuged at 10,500 × *g* for 11 min. The resulting pellet was resuspended in 12% Percoll, layered to Percoll gradient (40–23%) and centrifuged at 30,700 × *g* at 4 °C for 15 min. The mitochondrial layer was collected and washed twice using centrifugation. The final pellet was resuspended in the IB containing 0.02 mM EGTA. The mitochondrial protein concentration was determined using a biuret procedure with bovine serum albumin as the standard.

### Measurements of mitochondrial potential in isolated rat brain mitochondria

Safranine O (10 µM) was used as a membrane potential probe^45^. Fluorescence intensity at 580 nm (excitation at 520 nm) was measured with Victor3 multi-well fluorescence plate reader (Perkin Elmer). Mitochondrial protein concentration was 0.2 mg/ml. The medium for measurements contained 75 mM sucrose, 225 mM mannitol, 10 mM K-HEPES (pH 7.4), 0.02 mM EGTA, 1 mM KH_2_PO_4_. After a 4-min incubation, substrates of respiratory chain (5 mM glutamate, 2 mM malate and the 5 mM succinate) were added to produce the mitochondrial potential. Then the different concentrations of the compound or the same volume of vehicle (DMSO) were injected to the mitochondrial suspension. After 4 min, 12.5 µM CaCl_2_ was added to each probe to induce the depolarization of mitochondria which leads to the opening of mPTP. Results on mitochondrial membrane potential changes after calcium addition were presented as the mean ± SD where the mean is the maximum rate of change in fluorescence normalized between control probe and rate of change in fluorescence before calcium additions.

### Ionomycin-induced calcium overload in differentiated neuroblastoma SH-SY5Y cell culture

SH-SY5Y neuroblastoma cells were cultured in Dulbecco’s modified Eagle’s medium (DMEM) containing high glucose (25 mM), l-glutamine (2 mM), and sodium pyruvate (1 mM). This medium was supplemented with 10% (v/v) heat-inactivated fetal calf serum and 1% penicillin streptomycin. Cells were cultivated at 37 °C with 5% CO_2_ at saturated humidity in 96-well plates. The differentiation of SH-SY5Y cells was carried out in DMEM containing high glucose (25 mM), l-glutamine (4 mM), 1% P/S, and no sodium pyruvate. The medium was further supplemented with 10 µM all-trans retinoic acid before adding the medium to the cells. Differentiation lasted 4 days, on the 5th day the experiment was carried out. Cells were incubated with different concentrations of the test compound or an equal volume of the vehicle (< 1% of the whole volume of the medium under the layer of cells) and 3 µM ionomycin for 24 h. The cell viability was evaluated as the dehydrogenase activity with the 3-(4,5-dimethylthiazol-2-yl)-2,5-diphenyltetrazolium bromide (MTT) assay and the absorbance was measured at 570 nm using a Victor microplate reader (Perkin Elmer).

### Primary neuronal cell culture

Mixed cultures of hippocampal and cortical neurons and glial cells were prepared as described previously^17^ with modifications, from Sprague–Dawley rat pups 2–4 days post-partum (UCL breeding colony). Experimental procedures were performed in full compliance with the United Kingdom Animal (Scientific Procedures) Act of 1986 and with approval of the University College London Animal Ethics Committee. Hippocampi and cortex were removed into ice-cold PBS (Ca^2+^, Mg^2+^-free, Invitrogen, Paisley, UK). The tissue was minced and trypsinised (0.25% for 15 min at 37 °C), triturated and plated on poly-d-lysine-coated coverslips and cultured in Neurobasal A medium (Invitrogen, Paisley, UK) supplemented with B-27 (Invitrogen, Paisley, UK) and 2 mM l-glutamine. Cultures were maintained at 37 °C in a humidified atmosphere of 5% CO_2_ and 95% air, fed once a week and maintained for a minimum of 12 days before experimental use to ensure expression of glutamate and other receptors. Neurons were easily distinguishable from glia: they appeared phase bright, had smooth rounded somata and distinct processes, and laid just above the focal plane of the glial layer. Cells were used at 12–15 days in vitro (DIV) unless otherwise stated.

### Imaging [Ca^2+^]_c_ and mitochondrial membrane potential

Cortical neurons were loaded for 30 min at room temperature with 5 μM Fura-2 AM and 0.005% Pluronic in a HEPES-buffered salt solution (HBSS) composed (mM): 156 NaCl, 3 KCl, 2MgSO_4_, 1.25 KH_2_PO_4_, 2 CaCl_2_, 10 glucose and 10 HEPES, pH adjusted to 7.35 with NaOH. For simultaneous measurement of [Ca^2+^]_c_ and mitochondrial membrane potential (∆ψ_m_), Rh123 (1 µM, Molecular Probes) was added into the cultures during the last 15 min of the Fura-2 loading period, and the cells were then washed 3–5 times before experiment.

Fluorescence measurements were obtained on an epifluorescence inverted microscope equipped with a 20× fluorite objective. [Ca^2+^]_i_ and ∆ψ_m_ were monitored in single cells using excitation light provided by a Xenon arc lamp, the beam passing sequentially through 10 nm band pass filters centred at 340, 380 and 490 nm housed in computer-controlled filter wheel (Cairn Research, Kent, UK). Emitted fluorescence light was reflected through a 515 nm long-pass filter to a cooled CCD camera (Retiga, QImaging, Canada). All imaging data were collected and analysed using software from Andor (Belfast, UK). The Fura-2 or Fura-ff data have not been calibrated in terms of [Ca^2+^]_i_ because of the uncertainty arising from the use of different calibration techniques and were presented as 340/380 nm ratio. Accumulation of Rh123 in polarised mitochondria quenches the fluorescent signal in cytosol; in response to depolarisation the fluorescence signal is dequenched; an increase in Rh123 signal in the whole neuron therefore indicates mitochondrial depolarisation. We have normalised the signals between resting level (set to 0) and a maximal signal generated in response to the protonophore FCCP (1 μM; set to 100%).

### Imaging cytosolic and mitochondrial Ca^2+^

Cortical neurons were loaded for 30 min at room temperature with 5 μM Fluo-4 AM, x-rhod-1 AM and 0.005% Pluronic and confocal images were obtained using a Zeiss 710 CLSM using a 40× oil immersion objective. The 488 nm Argon laser line was used to excite Fluo-4 fluorescence which was measured at 505–550 nm. Illumination intensity was kept to a minimum (at 0.1–0.2% of laser output) to avoid phototoxicity and the pinhole set to give an optical slice of ~ 2 µm. For x-rhod-1 measurements the 563 nm excitation and 580–630 nm emission were used. All data presented were obtained from at least 5 coverslips and 2–3 different cell preparations.

### Toxicity experiments

For toxicity assays the cells were loaded simultaneously with 20 µM propidium iodide (PI), which is excluded from viable cells but exhibits a red fluorescence following a loss of membrane integrity, and 4.5 µM Hoechst 33342 (Molecular Probes, Eugene, OR), which labels nuclei blue, to count the total number of cells. Using phase contrast optics, a bright field image allowed identification of neurones, which look quite different to the flatter glial component and also lie in a different focal plane, above the glial layer. A total number of 600–800 neurones were counted in 20–25 fields of each coverslip. Each experiment was repeated four or more times using separate cultures.

### In vivo studies of the effectiveness of Tg-2113x

#### Animals

All animal procedures were carried out in accordance with the local regulations and approved by the Bioethics Committee of IPAC RAS (Approval No. 41, date 29 November 2019). 3 and 16-months-old male C57BL/6j mice used in the study. All animals were housed individually, under 12 h light–dark cycle (lights on: 7:00 a.m.) with food and water ad libitum, under constant controlled laboratory conditions (22 ± 1 °C, 55% humidity).

Mice were administered Tg-2113x and scopolamine in the vivarium from 8:30 to 9:00. Behavioral studies were carried out after at least 1-h acclimatization time to the experimental room, in the dark, from 9:00 to 18:00. All efforts were undertaken to minimize the potential discomfort of experimental animals.

The equipment of the “Centre for Collective Use of IPAC RAS” was used in this work.

### Study design

Tg-2113x was dissolved in dimethyl sulfoxide and sterile 0.9% saline (DMSO:NaCl = 1:20) and administered intraperitoneally and a volume of injection of 0.01 ml per 10 g of body weight. Scopolamine was diluted with sterile 0.9% saline and administered subcutaneously, 0.05 ml per 10 g of body weight. Mice were treated with the drugs for 5 consecutive days, while in the 3rd day mice were exposed to the fear conditioning test (Fig. 1A). The choice of the administration protocol and the doses (Tg-2113x—0.5 mg/kg/day and scopolamine—0.1 mg/kg/day) were based on pilot experiments (data not shown). Given that the experimenter is a contextual signal for animals^46^, all experiments were conducted by one person who was at the same place throughout the test.

In addition, Tg-2113x was investigated in the novel cage, dark–light box and Porsolt's tests to eliminate potential anxiety- and depressive-like effects.

### Fear conditioning test

In the fear conditioning paradigm, mice were trained with a 2 s foot-shock (0.5 mA, 50 Hz) by a shocker (Evolocus, Terrytown, NY, USA), which was delivered after a 2-min acclimatization period. The apparatus (Open Science, Russia) consisted of a transparent plastic cubicle (25 × 25 × 50 cm) with a stainless-steel grid floor (33 rods/2mmin diameter). After delivery of the current, the mouse was immediately placed back into the home cage. Twenty-four hours later, freezing behavior was scored in a 180-s recall of extinction session. The occurrence of freezing behavior was assessed every 10 s, and each 10-s period was assigned to a freezing or non-freezing period, and the percentage of time spent freezing was calculated. Immediately after a recall session, animals were exposed to a memory extinguishing procedure. Therefore, mice were left for another 7 min in the apparatus, so the total procedure of memory extinction was 10-min long. During this period, no foot shock was applied, and animals were free to explore the apparatus. Twenty-four hours later, freezing behavior was scored again in a 180-s recall of extinction session as in the previous trial and percentage of time spent in freezing was calculated^47^.

### Novel cage test

The novel cage test was performed to assess vertical exploratory activity in a new environment. Mice were introduced into a standard plastic cage (21 × 21 × 15 cm) filled with fresh sawdust. The number of exploratory rears each minute was counted for a 5-min period.

### Dark–light box

Mice were placed into the black compartment (15 × 20 × 25 cm) from which they could visit the light compartment (30 × 20 × 25 cm, illumination intensity 25 Lux). During a 5-min period, the latency of the first transition, time spent in the light compartment and the number of transitions between compartments were recorded^48^.

### Porsolt’s test

In the test, mice were placed in a transparent tank (Ø 17 cm) filled with water (+ 23 °C) for 6-min and scored for the duration of floating, as described elsewhere^48^.

### Statistical analysis

The in vitro data was analyzed using GraphPad Prism 7.00 software (San Diego, CA, USA) by 1-way ANOVA followed by the Dannett’s multiple comparisons test. The in vivo data was analyzed using GraphPad Prism 7.00 software (San Diego, CA, USA) by repeated measures (RM) 2-way ANOVA followed by the Sidak's multiple comparisons test, unpaired t-test with Shapiro–Wilk normality test or Mann–Whitney nonparametric test. The level of confidence was set at 95% (p < 0.05). Data are given as mean ± SEM or median with interquartile range or 25th and 75th percentile.

### Ethics approval

This study was performed in line with the principles of the Declaration of Helsinki and was carried out in compliance with the ARRIVE guidelines. All animal procedures were approved by the Bioethics Committee of IPAC RAS (Approval No. 41, date 29 November 2019).

## Results

### Tg-2112x and Tg-2113x did not change the amplitude of the acetylcholine-induced calcium signal in cortical neurons

**Figure 2:**
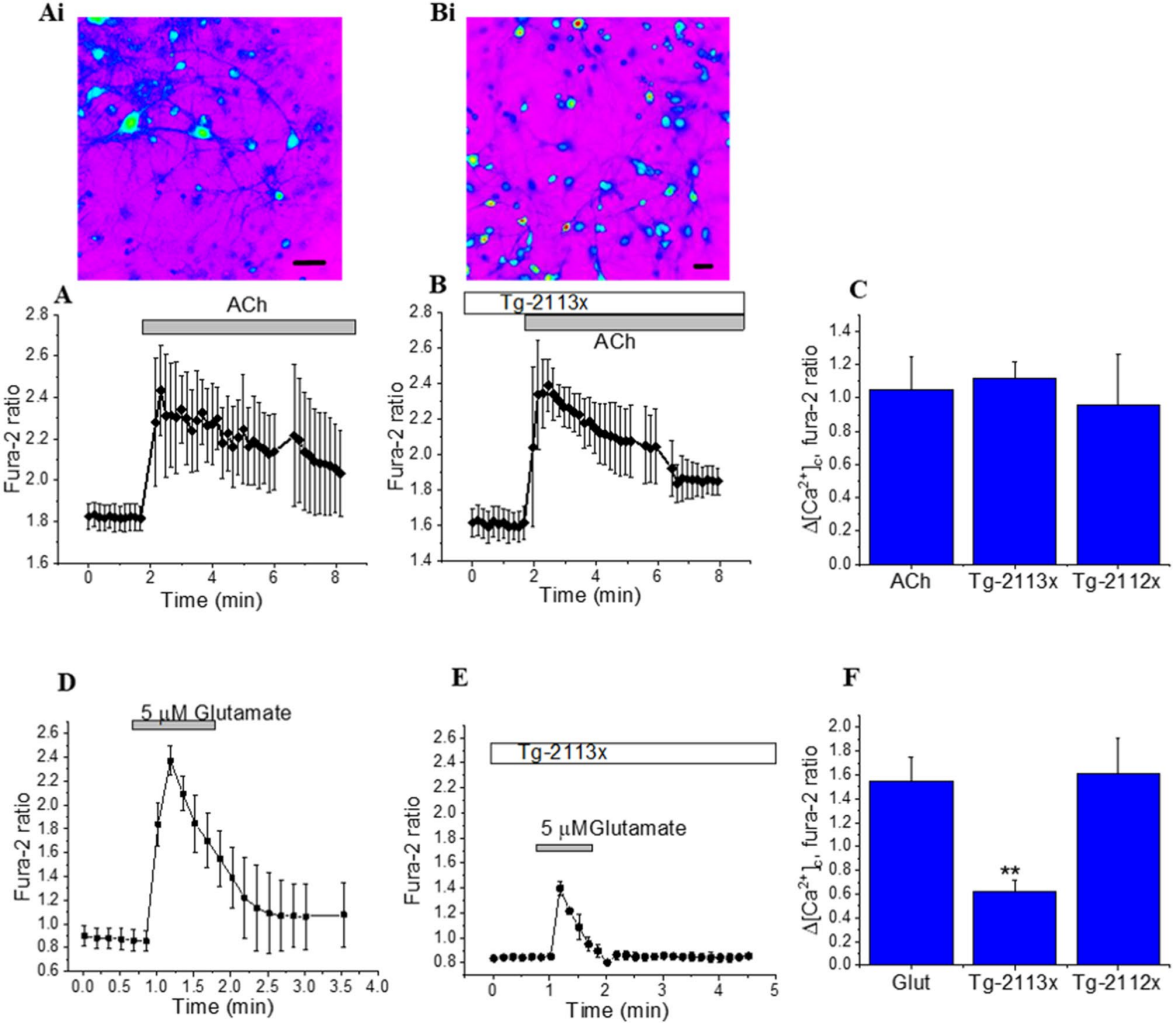
Effect of Tg-2113 on the amplitude of the acetylcholine- and glutamate-induced calcium signal. Effect of Ach (1 μM) on [Ca^2+^]_c_ of primary neurons in control (**A**) and in the presence of Tg-2113x (**B**) these data presented with typical images of fura-2 340/380 ratio with neurons and astrocytes (Ai. Bi). Bars on the images 20 μm. (**C**) Amplitudes of Ach-induced calcium elevation as changes in the Fura-2 ratio. Effect of 5 μM glutamate on [Ca^2+^]_c_ of primary neurons in control (**D**) and in the presence of Tg-2113x (**E**). (**F**) Summaries of the effects of Tg-2113x on glutamate-induced calcium signal. *p < 0.05; **< 0.01.

We have tested the effect of the compounds on the major receptors which were shown to be involved in the mechanism of pathology of neurodegeneration. Thus, 1 µM acetylcholine (Ach) induced a peak in [Ca^2+^]_c_ of primary cortical neurons (n = 155 cells; Fig. 2A)^49^. Pre-incubation of the cells with 0.5–5 µM Tg-2113x or 0.5–5 µM Tg-2112x had no effect on the number of neurons showing calcium signals or the amplitude of Ach-induced [Ca^2+^]_c_ changes (Fig. 2A–C).

### Tg-2113x partially inhibits glutamate-induced calcium signals in neurons

Transient application of 5 µM glutamate to the cortical co-culture induced a rise in [Ca^2+^]_c_ typical for this concentration in neurons but not in astrocytes (Fig. 2D). In agreement with previous data^50^, 0.5 µM Tg-2112x did not reduce the glutamate-induced calcium signal in neurons (n = 114 neurons; Fig. 1F). In contrast, pre-incubation of the cells with 0.5 µM Tg-2113x reduced the amplitude of the glutamate-induced calcium signal (n = 165 neurons; from 1.55 ± 0.2 Fura-2 ratio to 0.6 ± 0.07; p < 0.01; Fig. 2E,F). Thus, Tg-2113x partially inhibits glutamate-induced calcium signal that may be explained by a previously shown effect of this compound on NMDA receptors^51^.

### Tg-2112x and Tg-2113x have no effect on βA-induced calcium signal but reduced mitochondrial depolarization

**Figure 3:**
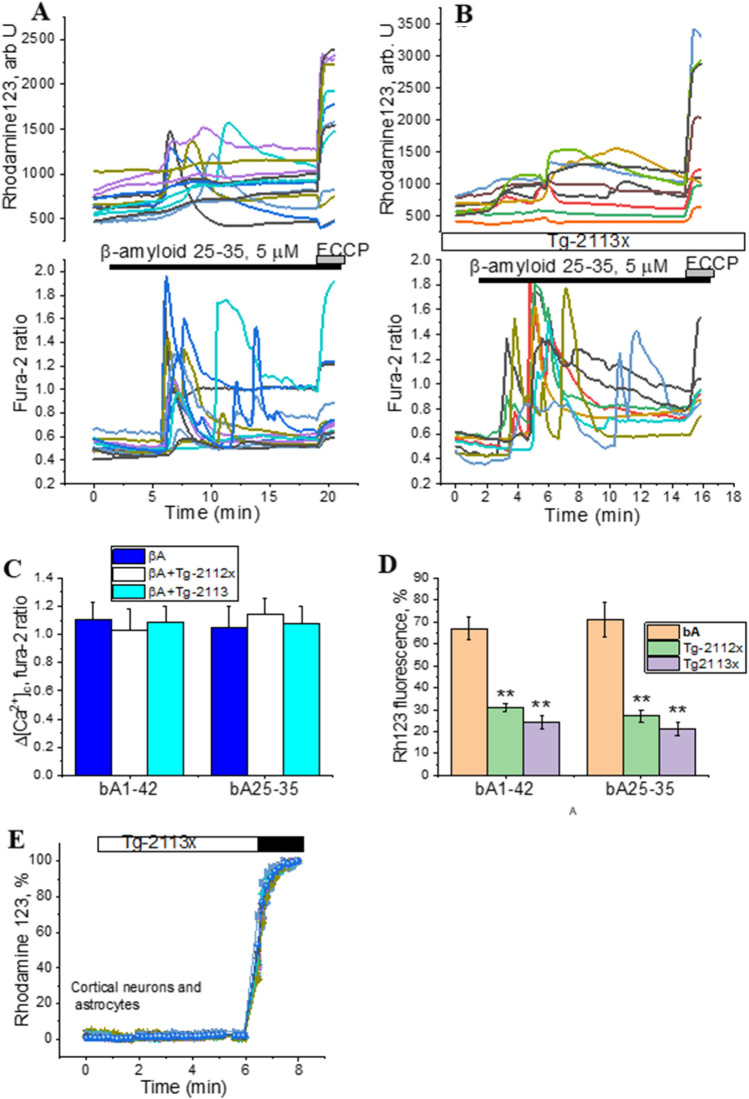
Effects of Tg2113x and Tg2112x on β-amyloid-induced calcium signal and mitochondrial depolarization in astrocytes from primary cortical co-cultures. Simultaneous measurement of β-A 25–35 (5 μM)-induced changes in [Ca^2+^]_c_ (fura-2 ratio) and mitochondrial membrane potential (Rhodamine123) in cortical astrocytes from the co-culture with neurons without Tg2113x (**A**) or after incubation with Tg2113x (**B**). Effects of Tg2112x and Tg2113x on the amplitude of βA 25–35-induced calcium signal in astrocytes (fura-2 ratio) (**C**) or mitochondrial membrane potential (% of Rhodamine123 fluorescence) (**D**). E- Tg2113x has no effect of Δψm (Rhodamine123) primary neurons and astrocytes. 1 μM FCCP was added in the end of experiments for calibration of signal. *p < 0.05; **< 0.01.

Application of the full peptide βA 1–42 (1 µM) or short peptide βA 25–35 (5 µM) to primary cultures induced the previously described calcium responses typical for these peptides in primary astrocytes after 5-10 min, but not in neurons from the same co-culture^4,52^ (Fig. 3A,C). In agreement with previously published data^13^, simultaneous measurements of Fura-2 and mitochondrial membrane potential (ΔΨm) with Rhodamine 123, showed the βA-induced loss of ΔΨm in astrocytes, with a profound and variable shape of the signal (Fig. 3A,D). Previously, we had shown that this type of signal is induced by oligomeric β-amyloid, and we did not additionally investigated the state of the aggregation of βA^53^. Pre-incubation of the primary co-culture of neurons and astrocytes for 10 min before the experiment with 0.5 µM Tg-2113x (N = 7 experiments) or 0.5 µM Tg-2112x (N = 6 experiments) did not change the effect of βA 25–35 or βA 1–42 on [Ca^2+^]_c_ elevation in astrocytes (Fig. 3B,C). However, both -Tg2112x and Tg-2113x significantly reduced the effect of βA 1–42 and βA 25–35 on mitochondrial membrane potential in astrocytes (Fig. 2B,D). Thus, Tg-2112x reduced mitochondrial depolarization from 67 ± 5% (βA 1–42, n = 126 astrocytes) to 31 ± 2% (n = 111 astrocytes) and from 71 ± 8% (βA 25–35, n = 99 astrocytes) to 27 ± 3% (n = 109; p < 0.01; Fig. 3D). Tg-2113x did also effectively reduce the action of βA1-42 on ΔΨm to 24 ± 3% (n = 143, p < 0.01) and βA 25–35 to 21 ± 3% (n = 121; p < 0.01; Fig. 3B–D). βA-induced mitochondrial depolarization is dependent on the overproduction of reactive oxygen species and mitochondrial calcium uptake^8,54^. Importantly, application of Tg-2113x had no acute effect on mitochondrial membrane potential of neurons and astrocytes (N = 4 experiments; Fig. 3E).

### Tg-2113x decreases βA-induced mitochondrial calcium uptake

**Figure 4:**
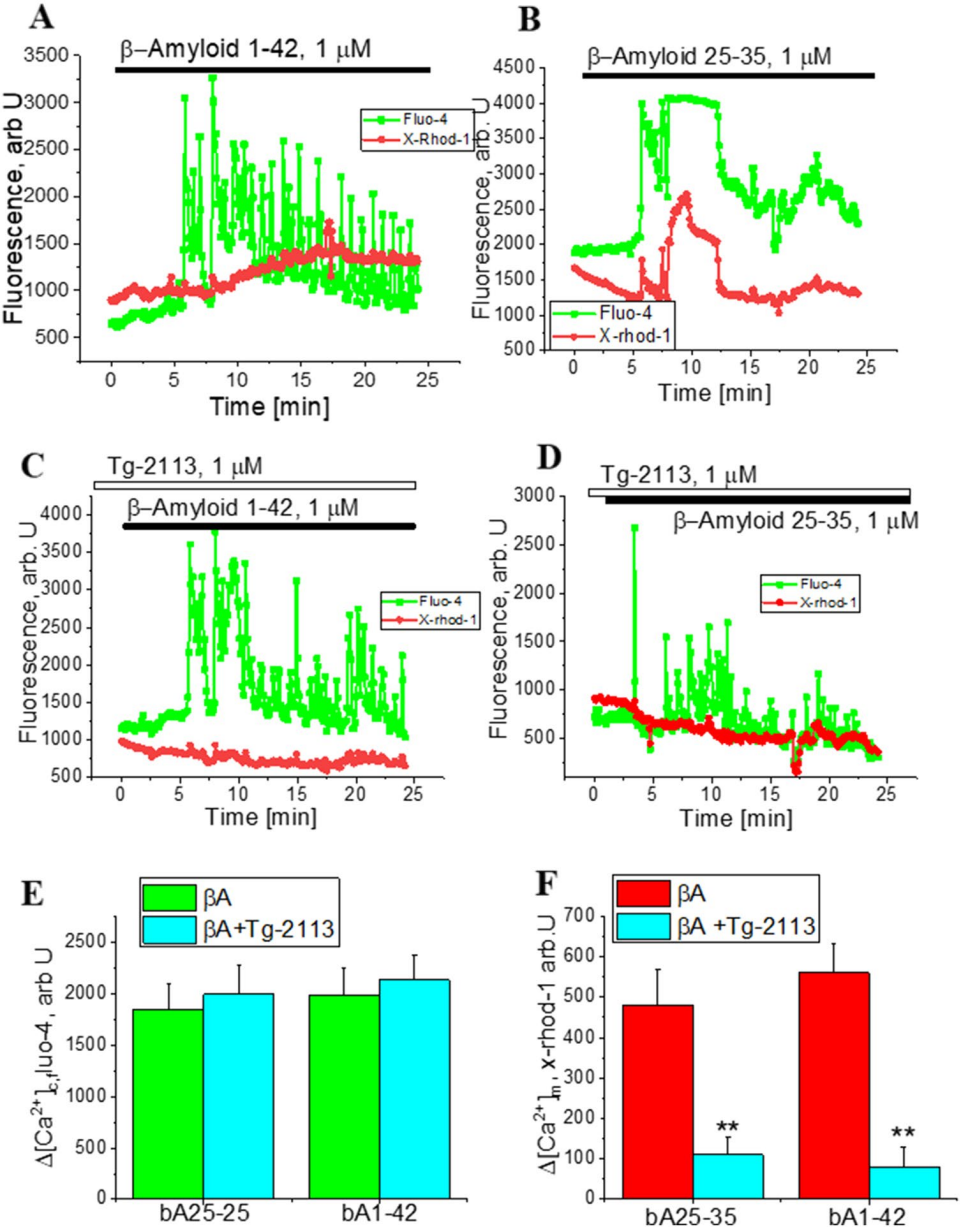
Tg-2113x inhibits mitochondrial calcium uptake but not β-Amyloid-induced calcium signal in cytosol. 1 μM βA_(1–42)_ (**A**) or 5 μM βA_(25–35)_ (**B**) induce an increase in cytosolic (Fluo-4) and mitochondrial calcium (x-Rhod-1). Incubation of the cells with Tg-2113x did not change βA_(25–35)_-induced cytosolic calcium signal but reduced mitochondrial calcium uptake (**C**, **D**). (**E**) Amplitude of βA_(25–35)_-induced changes in Fluo-4 signal with and without incubation with Tg-2113x. (**F**) Changes in mitochondrial Ca^2+^ (x-Rhod-1) after application of βA_(25–35)_ or βA_(1–42)_ in control and after incubation with Tg2113x. *p < 0.05; **< 0.01.

In order to identify the effect of Tg-2113x on mitochondrial calcium in the time of application of βA we used the mitochondrial calcium indicator X-rhod-1 co-loaded with indicator for cytosolic Ca^2+^ fluo-4. Similarly to Fura-2 measurements (Fig. 3), application of 1 µM βA 1–42 or 5 µM βA 25–35 to the co-culture of cortical neurons and astrocytes induced sporadic changes in [Ca^2+^]_c_ of astrocytes (n = 88 astrocytes for βA 1–42; n = 96 astrocytes for βA25-35; Fig. 4A,B,E). Preincubation of the cells with 0.5 µM Tg-2113x did not change the amplitude of βA 1–42 or βA 25–35-induced calcium signal in astrocytes (Fig. 4C). However, βA-induced cytosolic calcium signal activates mitochondrial calcium uptake (Fig. 4A,B,F) which was inhibited by incubation of the cells with 0.5 µM Tg-2113x (Fig. 4C,D,F). Thus, Tg-2113x inhibits mitochondrial calcium uptake in astrocytes, which protects cells against βA-induced mitochondrial depolarization.

### Tg-2113x and Tg-2112x protect neurons and astrocytes against βA-induced toxicity

**Figure 5:**
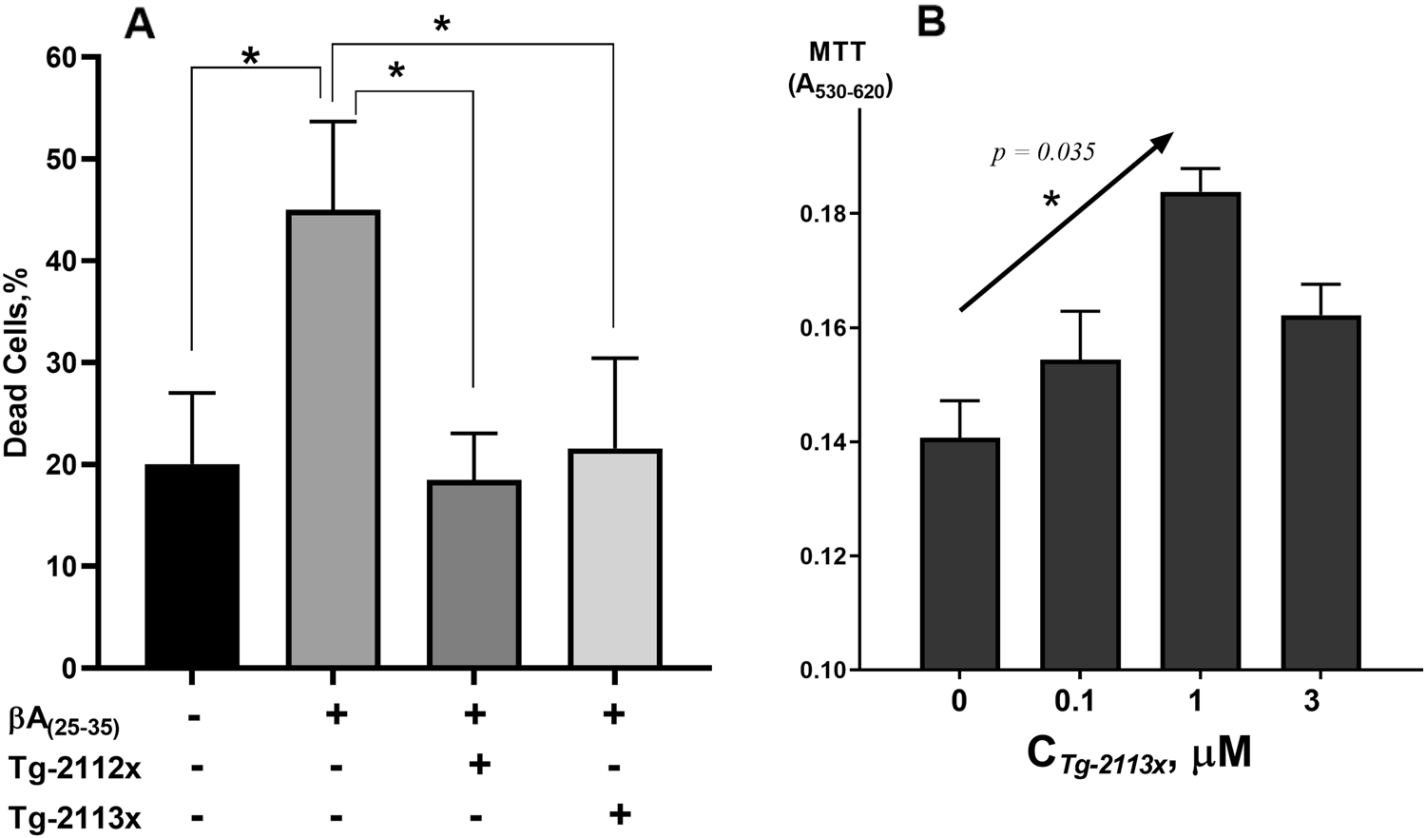
Tg-2112x and Tg-2113x protect cells against βA- and ionomycin-induced toxicity. (**A**) Tg-2112x and Tg-2113x reduced the percentage of βA_(25–35)_-induced cell death in co-cultures of neurons and astrocytes, *p < 0.05; (**B**) Tg-2113x protects differentiated SH-SY5Y neuroblastoma cells against ionomycin (3 μM)-induced cell toxicity (arrow show the test for linear trend as post-hoc testing after ANOVA, *p < 0.05).

24 h incubation of the cortical co-culture of neurons and astrocytes with 5 µM βA 25–35 induced a significant increase in the number of dead cells (from 20 ± 6% in control, N = 5; to 45 ± 8%, N = 5; p < 0.005; Fig. 5A). Incubation of the cells with 0.5 µM Tg-2112x or 0.5 µM Tg-2113x effectively inhibited βA-neurotoxicity. Thus, Tg-2112x reduced the number of dead cells to 18 ± 3%, N = 5, and Tg-2113x to 21 ± 4%, N = 5 (Fig. 5A). Thus, Tg-2113x and Tg-2112x prevent βA-induced mitochondrial calcium overload and mitochondrial depolarization, leading to neuroprotection.

β-Amyloid neurotoxicity is induced indirectly through its effect on astrocytes^55^. In order to study a direct effect of calcium-induced cell death on homogeneous cells (neuronal model) we used SH-SY5Y cells. Thus, the neuroprotective effect of Tg-2113x was primarily investigated in the calcium overload model of neurodegeneration on differentiated SH-SY5Y neuroblastoma cells. This model allows to verify non-receptor induced neuroprotective effects, due to the fact that SH-SY5Y have no functional glutamate receptors, and the use of the calcium ionophore ionomycin to induce calcium overload. Tg-2113x alone had no effect the viability of the cells at all studied concentrations, but at concentrations of 30 μM and above caused a decrease in cell viability. Calcium overload induced by incubation of the cells with ionomycin for 24 h led to more than 40% SH-SY5Y neuronal death. When Tg-2113x was present, the viability of the cells increased and was dose-dependent and significant (Fig. 5B). However, at 3 μM the protective effect begins to decrease, which may be due to the onset of the manifestation of the compound's own toxicity.

### Tg-2113x inhibits calcium uptake in mitochondria of permeabilized neurons and astrocytes

**Figure 6:**
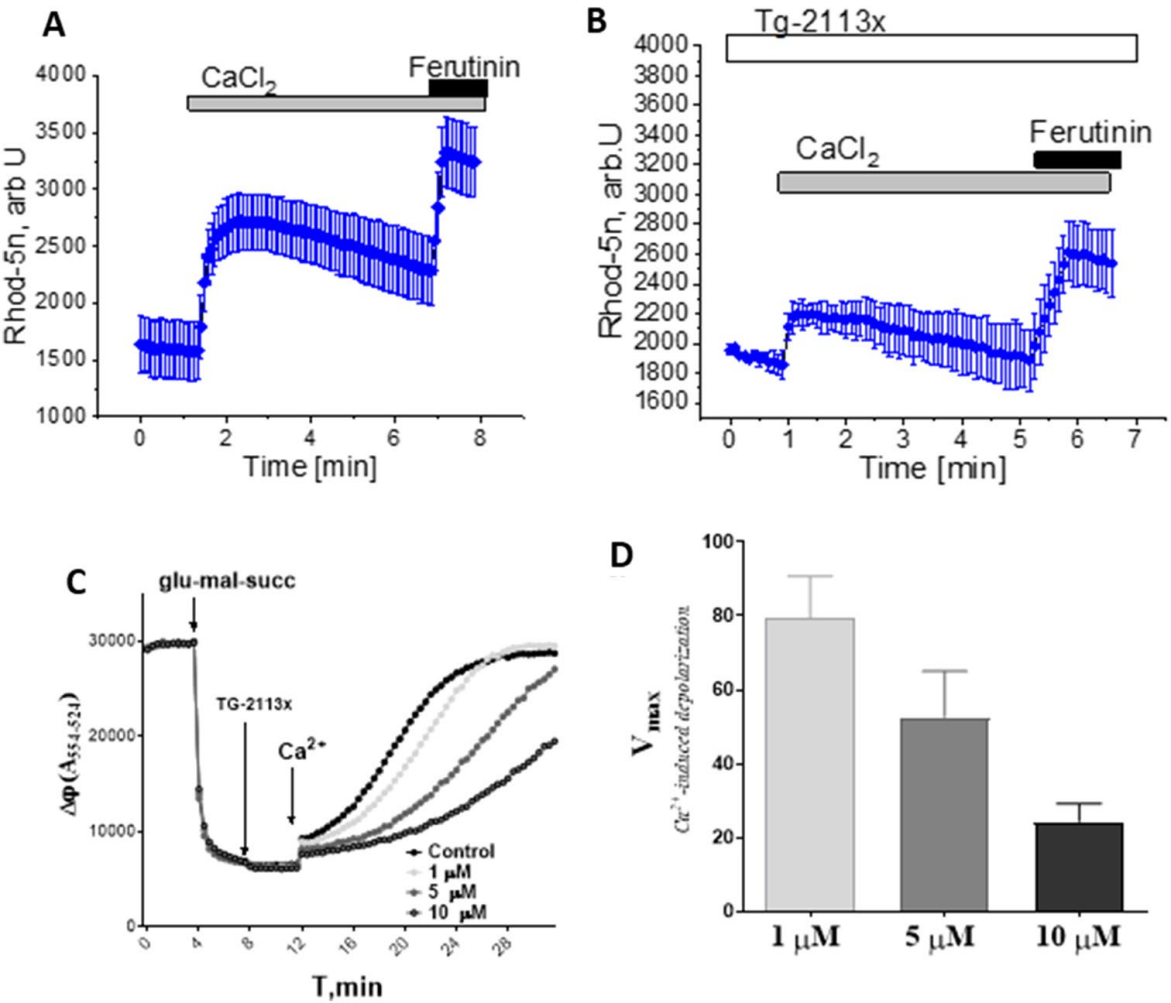
Effect of Tg-2113x on permeabilized cells and isolated mitochondria: application of 100 nM–5 µM calcium to mitochondria of permeabilized neurons induced a rise in [Ca^2+^]_m_ in control (**A**, n = 5 experiments), but not in 0.5 µM Tg-2113x (**B**, n = 4 experiments)-treated permeabilized neurons. Ferutinin at the end of the experiment confirms that mitochondria are still intact; (**C**) typical isolated mitochondria potential estimation experiment, (**D**) mitochondrial membrane potential changes after calcium addition as the maximum rate of change in fluorescence normalized between control probe and rate of change in fluorescence before calcium additions.

To confirm that the effects seen in the experiments with intact neurons are directly related to the changes in the activity of the mitochondrial Ca^2+^ transport we measured Ca^2+^ uptake in mitochondria of permeabilized cells. Application of buffered Ca^2+^ (0.2 μM and 1 µM, n = 6 experiments; Fig. 6A) increased fluorescence of the mitochondrial calcium marker Rhod-5 N. Addition of the same concentrations of CaCl_2_ to permeabilized neurons and astrocytes in the presence of Tg-2113x (0.5 µM; N = 5 experiments) significantly reduced the effect on [Ca^2+^]_m_. Importantly, these mitochondria were still viable, because the electrogenic ionophore Ferutinin^56,57^ induced a further increase in mitochondrial calcium (Fig. 6B). Thus, Tg-2113x inhibits physiological influx into mitochondria while an alternative transport, such as the one induced by the electrogenic calcium ionophore Ferutinin is still able to produce an increase of Ca^2+^ in these mitochondria.

### Tg-2113x decreases the Ca^2+-^induced depolarization of rat brain mitochondria and protects cells from calcium overload

Previously we showed that the derivative of tetrahydrocarbazole and aminoadamantane (Tg-2112x) effectively inhibits the opening of mPTP in brain mitochondria and increases their calcium retention capacity^17^. The influence of the derivative of tetrahydrocarbazole and dimethylaminoadamantane (Tg-2113x) on calcium-induced depolarization was also studied. We observed that this compound did not influence the mitochondrial potential at all studied concentrations, but in concentrations from 1 µM and higher decreased the calcium-induced depolarization of mitochondria (Fig. 6C,D). This allows us to conclude that Tg-2113x, like the related compound Tg-2112x can delay mPTP opening.

### In vivo studies of the effectiveness of Tg-2113x

#### Tg-2113x neutralizes scopolamine-induced amnesia in young mice, but does not affect the memory of the Non-scopolamine animals

**Figure 7:**
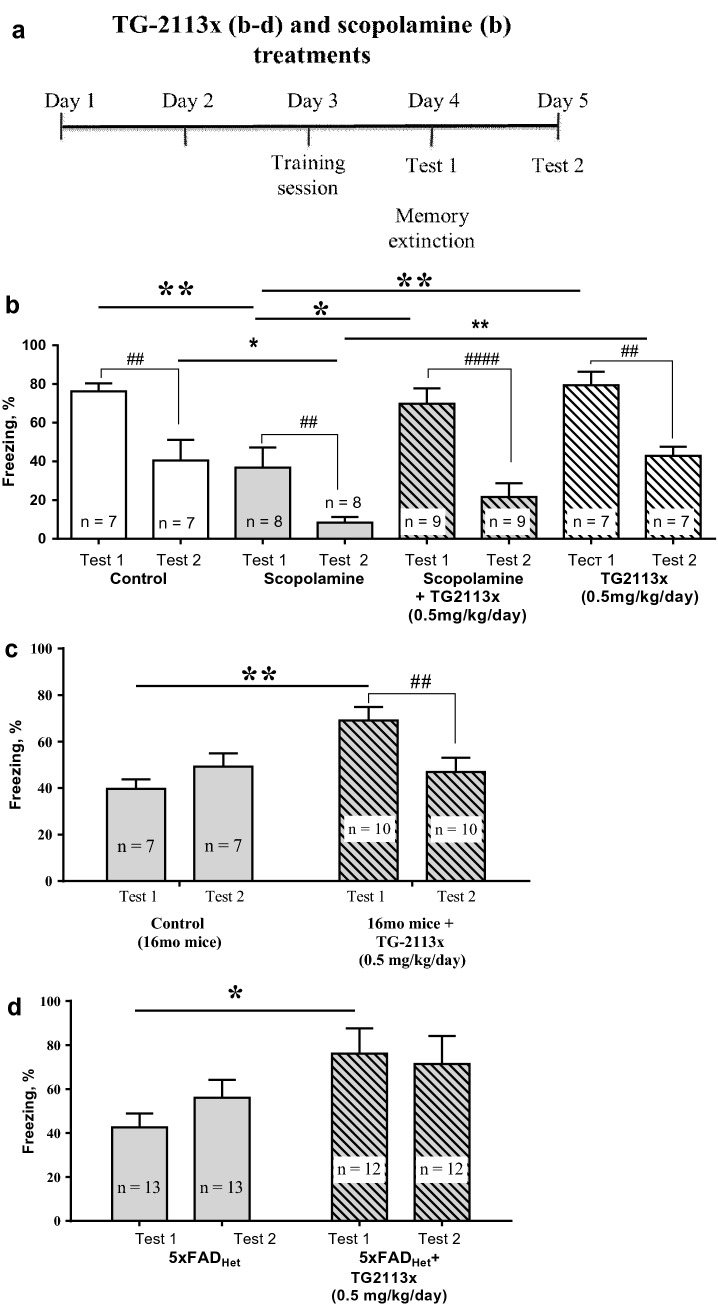
Antiamnestic properties of Tg-2113x in the Fear conditioning test: (**a**) timetable of experiments, mice were treated with ScA and Tg2113x for 5 consecutive days; (**b**) Tg-2113x neutralizes scopolamine-induced amnesia in young mice, does not affect the memory of the Non-Scopolamine group of animals, RM two-way ANOVA (F_interaction_ (3, 27) = 0.9459, P = 0.4323; _Ftest1–test2_ (1, 27) = 70.83, P < 0.0001; F_treatment_ (3, 27) = 8.321, P = 0.0004; F_Subjects (matching)_ (27, 27) = 1.886, P = 0.0520); (**c**) Tg-2113x improves contextual memory and its extinction in 16-months-old mice, RM two-way ANOVA (F_interaction_ (1, 14) = 10.5, P = 0.0059; _Ftest1-test2_ (1, 14) = 1.678, P = 0.2161; F_control-TG-2113x_ (1, 14) = 4.442, P = 0.0536; F_Subjects (matching)_ (14, 14) = 1.731, P = 0.1580); (**d**) Tg-2113x prevents impaired fear conditioning, but not fear extinction, in 9-months-old 5xFAD mice, RM two-way ANOVA (F_interaction_ (1, 23) = 2.158, P = 0.1554; _Ftest1–test2_ (1, 23) = 0.5, P = 0.4866; F_5xFAD-(5xFAD-TG-2113x)_ (1, 23) = 3.872, P = 0.0613; F_Subjects(matching)_ (23, 23) = 4.016, P = 0.0007). *, *p < 0.05; **, **, ^##^p < 0.01; ^####^p < 0.0001.

In the model of scopolamine-induced amnesia in 3-months-old C57Bl/6j mice (Fig. 7a), we found a significant group difference in the freezing behavior (Fig. 7b). RM two-way ANOVA followed by Sidak's multiple comparisons showed that associative learning (freezing in the test 1) was significantly different between control and scopolamine (ScA)-treated mice (P = 0.0069), between only ScA and ScA simultaneously with Tg-2113x treated mice (P = 0.0229), between ScA and Tg-2113x treated mice (P = 0.0027). But extinction (freezing in the test 2) was only significantly different between control and ScA treated mice (P = 0.0497) and between ScA and Tg-2113x treated mice (P = 0.0267).

In vivo experiments showed a decreased freezing in scopolamine-treated mice (Fig. 7b), suggesting a violation of the process of remembering a dangerous context. Tg-2113x administration prevented the scopolamine-induced decrease in freezing, i.e. prevents memory impairment. Tg-2113x administration to the “Non-Scopolamine” group of mice did not change the freezing behavior (Fig. 7b), what we regarded as no influence on normal memory processes.

All groups showed significant decreased freezing during test 2 in comparison to the test 1 (P = 0.0015 for control mice; P = 0.0015 for Tg-2113x treated mice; P = 0.0028 for ScA-treated mice; P < 0001 for ScA and TG-2113x-treated mice), suggesting that neither treatment alters memory extinction, and it is impossible to conclude on the impact of Tg-2113x on this form of memory in young and/or scopolamine-treated mice. A lack of changes of extinction in scopolamine-treated mice is consistent with other authors work^58^.

The results confirm the neuroprotective effects of Tg-2113x and suggest that it does not improve cognitive function of young healthy mice without neurodegenerative pathology.

#### Tg-2113x improves contextual memory and its extinction in 16-months-old mice

In the aged mice RM two-way ANOVA followed by Sidak's multiple comparisons showed that freezing significantly distinguishes between control and Tg-2113x-treated mice in test 1 (P = 0.0022, Fig. 7c) not in test 2, moreover freezing was significantly different between test 1 and test 2 Tg-2113x-treated mice (P = 0.0081, Fig. 7c), but not for control 16-month-age mice (P = 0.3851, Fig. 7c).). In test 1, Tg-2113x-treated mice spent significantly longer percent of time in freezing than vehicle-treated mice, which suggests that Tg-2113x improved contextual memory of 16-months-old mice. While in the control group, percent of freezing did not differ between tests 1 and 2 (Fig. 7c), Tg-2113x-treated mice spent significantly less time in freezing in test 2, demonstrating effective fear extinction. Based on these data, we hypothesized that Tg-2113 can restore the age-impaired decline in memory extinction and contribute to greater plasticity of cognitive processes with age.

#### Tg-2113x prevents impaired fear conditioning, but not fear extinction, in 9-months-old 5xFAD mice

In heterozygous (Het) 5xFAD mice, the model of cerebral amyloidosis, RM two-way ANOVA followed by Sidak’s multiple comparisons showed that freezing significantly distinguished between 5xFAD mice and Tg-2113x-treated 5xFAD mice in the test 1 (P = 0.039, Fig. 7d) not in test 2 (P = 0.4744, Fig. 7d), without difference in freezing between test 1 and test 2 (P = 0.2430 and P = 0.8419, accordingly, Fig. 7d).

The results suggest that Tg-2113x prevents Aβ-induced impaired fear conditioning, but not fear extinction, in 5xFAD mice.

#### Tg-2113x does not affect the exploratory, anxiety-, and depressive-like behaviour of young mice

**Figure 8:**
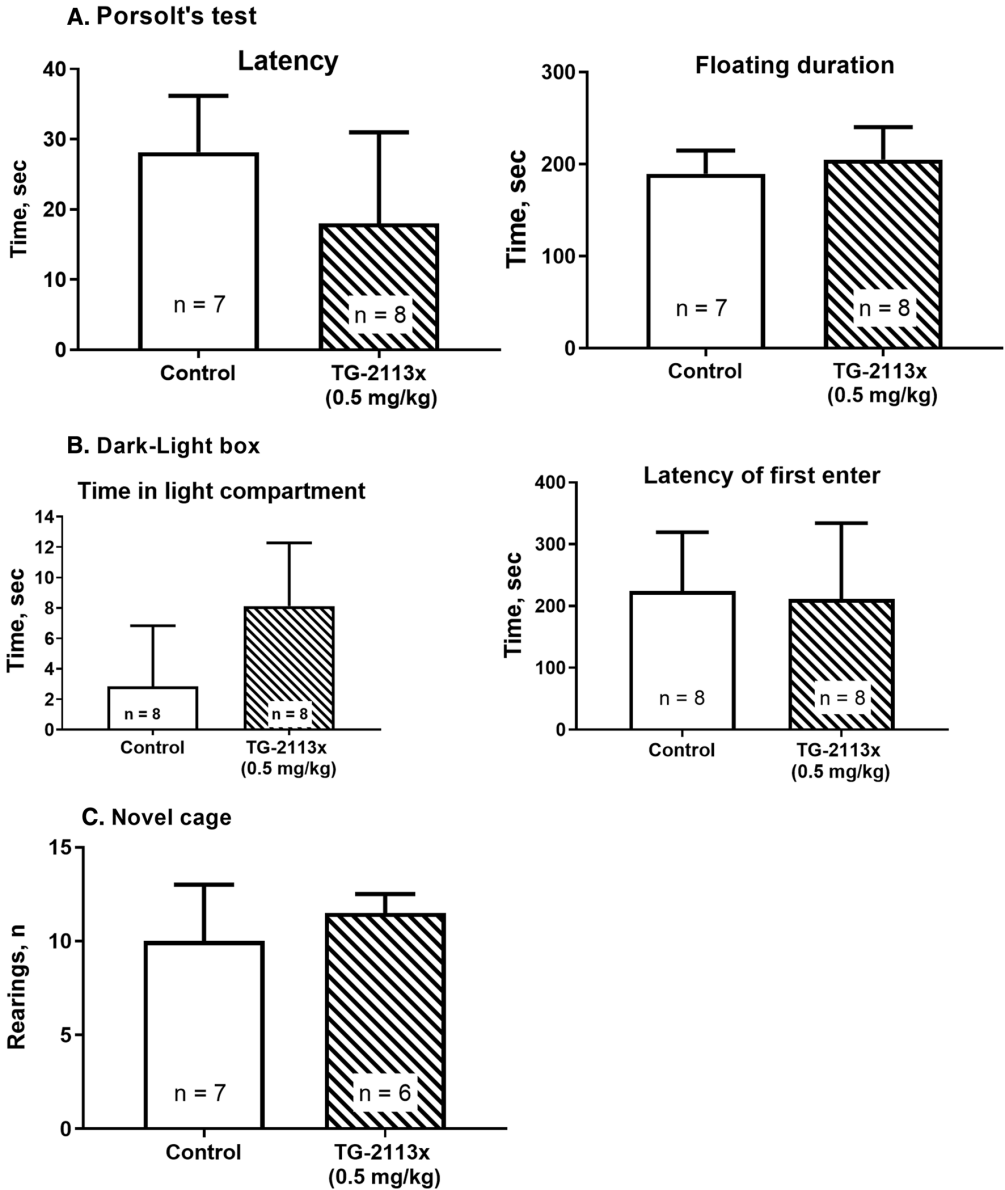
Tg-2113x does not affect the depressive-like (**A**), anxiety-like (**B**) and exploratory (**C**) behavior of young (3 mounths-old) mice.

To further evaluate the effect of Tg-2113x in the behavior of mice that could influence the results and discard potential undesirable effects, we performed additional tests. The possible effect of Tg-2113x on depressive-like behavior of mice was investigated with the Porsolt's test. There was no difference in the latency and floating duration between Tg-2113x- and vehicle-treated groups (t = 1.809, df = 14, P = 0.0920 and t = 0.9651, df = 14, P = 3509, respectively, unpaired t-test, Fig. 8A). Tg-2113x did not alter anxiety-like behavior of mice in the dark–light box), as it was shown by no difference in the latency of the first exit in the light apartment and time spent there, between Tg-2113x- and vehicle-treated groups (t = 1.127, df = 13, P = 0.28 and t = 0.2322, df = 14, P = 0.8197, respectively, unpaired t-test, Fig. 8B). Moreover, animals were scored for exploratory rears in the novel cage test. There was not a significant difference in exploratory rears in the test for the experimental groups (control: M = 10.0 (7.0; 13.0), TG-2113x: M = 11.5(10.75; 12.50); p = 0.2762; Mann–Whitney test, Fig. 8C).

Thus, it can be suggested, Tg-2113x does not affect the general behavior of young healthy mice.

## Discussion

Dementia has a multifactorial pathogenesis, and no model includes all disease aspects, but only partially mimics pathological and/or etiologic factors^59,60^. Therefore, we believe that to comprehensively study the new potential treatments is critical to use numerous and diverse models of the disease. Following this idea, this study was performed in various mouse models of neurodegenerative disease, induced by age (16-months-old C57Bl/6j mice), cholinergic dysfunction (scopolamine-induced amnesia in 3-months-old C57Bl/6j mice) or Aβ plaques (9-months-old 5xFAD mice). Mice were exposed to tests to assess cognitive function, cognitive plasticity and general behavior. It was shown that 5 days dosage with 0.5 mg/kg/day with Tg-2113x improves cognitive function of mice in models of different etiology of dementia but does not affect the memory and general behavior of young healthy animals. The last finding suggests a decreased risk of undesirable side-effects of Tg-2113x in the clinic.

The protective effect of Tg-2113x in all dementia models could be mostly explained by its ability to limit the calcium uptake by mitochondria. Effect of Tg-2113x on glutamate receptors may also have an implication, but it could not be the solely mechanism of protection. Thus, in the model of scopolamine-induced amnesia in 3-months-old C57Bl/6j mice with cholinergic dysfunction, inhibition of glutamate receptors may not have a significant effect and Tg-2113x has no effect on Ach-induced calcium signal (Fig. 1). The data with Aβ plaques (9-months-old 5xFAD mice) are also in agreement with the effects of Tg-2113x on Aβ -induced calcium signal, mitochondrial membrane potential and mitochondrial calcium and suggested that inhibition of mitochondrial calcium uptake could be a major mechanism for cell protection.

Both in humans and animals, solving a particular problem is a choice between actual and irrelevant information at the moment. This choice is carried out by controlled inhibitory processes that suppress irrelevant information. In aged persons or patients with dementia, disruption of these processes leads to disorder of memory extinction and a competition between information, and therefore, difficulties in solving a problem^61^. Therefore here, we chose the protocol of fear conditioning test that includes the session of memory extinction. In comparison to young, aged mice show a decrease in memory function, as reported here and in other works^62^. But unlike our study authors usually used animals on six months older than we did, and our results suggest that aged-related cognitive dysfunctions can already be detected in 16-months-old C57Bl/6j males. Furthermore, as far as we know, age changes in memory extinction have not been evaluated in classical Pavlovian conditioning, and our work is probably one of the first to show an impairment of memory extinction in aged mice. In our view, this impairment means a disorder of the controlled processes that suppress irrelevant information that was mentioned above. Another model which is designed to mimic age-related dementia, the scopolamine-induced amnesia^63^, did not induce changes in fear extinction of mice, so the 16-months-old mice can be proposed as a well valid model of age-induced dysfunction of cognitive plasticity. Tg-2113x was shown to improve memory conditioning and extinction in aged mice, implying recovery of age-defected cognitive plasticity.

During memory extinction, two processes occur—the consolidation of and the suppression of irrelevant one—relating to the same issue. However, the primary memory remains, and this distinguishes extinction from forgetting^64^. The suppression of irrelevant memory can be realized via the GABAergic system. Indeed, GABA antagonists were shown to impede extinction, and GABA agonists facilitate it^65^. Moreover, memory extinction is associated with changes in the expression of genes associated with the GABAergic system. For example, the decrease of the mRNA level of α2 and β2 subunits of GABA receptors, the glutamate decarboxylase, that catalyzes reaction of glutamate to GABA, and the GABA transporter were observed^66^.

At the same time, both the formation of memory and its extinction involve the glutamatergic system. While administration of NMDA receptor antagonists blocks the extinction of conditioned fear, NMDA agonists facilitate it^67^. There is an evidence that the GluN2B subunit of the NMDA receptor is specifically involved in this process^66^. Biochemical experiments have also shown that extinction is associated with a decrease in the expression of AMPA receptors (GluA1 and GluA2)^68^. As NMDA receptors is one of the Tg-2113x targets, it can be suggested that its positive effect on cognitive plasticity of mice is mediated by the modulation of the glutamatergic system. On the other hand, prevention of the glutamate-induced calcium influx into neurons and the mitoprotective action of Tg-2113x may be the basis of the neuroprotective effect in experiments in vivo, especially under conditions of scopolamine-induced amnesia.

The effectiveness of Tg-2113x in scopolamine-treated animals, and in aged mice can be explored by its ability to modulate the cholinergic system. As we have shown Tg-2113x does not affect the acetylcholine-induced activation of neuronal calcium uptake, but Tg-2113x inhibits butyrylcholinesterase, that catalyzes the hydrolysis of acetylcholine, thereby increasing the choline level essential for memory formation^18,51^. The efficacy of selective inhibitors of butyrylcholinesterase as cognitive-stimulating compounds has already been demonstrated by other authors^69^. Moreover, in models of scopolamine-induced amnesia and 5xFAD mice the previously proposed neuroprotective functions of Tg-2113x were proven.

## Conclusion

Tg-2112x and Tg2113x significantly reduce mitochondrial calcium uptake without alteration of the mitochondrial membrane potential. Both compounds reduced the mitochondrial calcium uptake and protected cells against β-amyloid mitochondrial depolarisation and cell death. Using three various mouse models of neurodegenerative disease, induced by age (16-months-old C57Bl/6j mice), cholinergic dysfunction (scopolamine-induced amnesia in 3-months-old C57Bl/6j mice) or amyloidosis (9-months-old 5xFAD mice) we have found a protective effect of Tg2113x against dementia, which also highlights the importance of mitochondrial calcium uptake in the pathogenesis of dementia. Our data suggests that a reduction in the mitochondrial calcium uptake can be used as a potential therapeutic strategy against neurodegeneration and dementia, and Tg2113x can be used as a promising lead-compound for developing on this background a novel generation of disease-modifying neuroprotective agents.

## Data Availability

All data supporting the conclusions of this manuscript are provided in the text and figures.
